# Cellular responses in the FGF10‐mediated improvement of hindlimb regenerative capacity in *Xenopus laevis* revealed by single‐cell transcriptomics

**DOI:** 10.1111/dgd.12795

**Published:** 2022-06-20

**Authors:** Nodoka Yanagi, Sumika Kato, Taro Fukazawa, Takeo Kubo

**Affiliations:** ^1^ Department of Biological Sciences, Graduate School of Science The University of Tokyo Tokyo Japan

**Keywords:** FGF10, hindlimb, regeneration, single‐cell RNA sequencing, *Xenopus laevis*

## Abstract

*Xenopus laevis* tadpoles possess regenerative capacity in their hindlimb buds at early developmental stages (stages ~52–54); they can regenerate complete hindlimbs with digits after limb bud amputation. However, they gradually lose their regenerative capacity as metamorphosis proceeds. Tadpoles in late developmental stages regenerate fewer digits (stage ~56), or only form cartilaginous spike without digits or joints (stage ~58 or later) after amputation. Previous studies have shown that administration of fibroblast growth factor 10 (FGF10) in late‐stage (stage 56) tadpole hindlimb buds after amputation can improve their regenerative capacity, which means that the cells responding to FGF10 signaling play an important role in limb bud regeneration. In this study, we performed single‐cell RNA sequencing (scRNA‐seq) of hindlimb buds that were amputated and administered FGF10 by implanting FGF10‐soaked beads at a late stage (stage 56), and explored cell clusters exhibiting a differential gene expression pattern compared with that in controls treated with phosphate‐buffered saline. The scRNA‐seq data showed expansion of *fgf8‐*expressing cells in the cluster of the apical epidermal cap of FGF10‐treated hindlimb buds, which was reported previously, indicating that the administration of FGF10 was successful. On analysis, in addition to the epidermal cluster, a subset of myeloid cells and a newly identified cluster of *steap4*‐expressing cells showed remarkable differences in their gene expression profiles between the FGF10‐ or phosphate‐buffered saline‐treatment conditions, suggesting a possible role of these clusters in improving the regenerative capacity of hindlimbs via FGF10 administration.

## 1 INTRODUCTION

Appendage regenerative capacity varies among vertebrates. Amphibians have long been studied as a model for the study of organ regeneration as they possess high limb regenerative capacity. Urodele amphibians such as salamanders retain the ability to fully reconstruct their lost parts of limbs, including the complete shape, pattern, and function, whereas anuran amphibians such as frogs demonstrate differential limb regenerative capacity depending on their developmental stage (Simon & Tanaka, 2013). In *Xenopus laevis* tadpoles, high regenerative capacity is observed prior to metamorphosis of their hindlimbs. Tadpoles at early developmental stages (stages ~52–54) exhibit complete limb bud regeneration after amputation, as do urodele amphibians. However, the capacity gradually declines as the developmental stage proceeds, with tadpoles at advanced developmental stages regenerating fewer digits (stage ~56), and even later (stage ~58 or later) forming only a simple cartilaginous spike without digits or joints after amputation (Dent, 1962).

Previous studies have tried to induce limb regeneration in *X. laevis* after metamorphosis by progenitor cell transplantation (Lin et al., 2013), activation of key molecular pathways (Lin et al., 2013; Satoh et al., 2005; Yakushiji et al., 2008; Yokoyama et al., 2001; Zhang et al., 2018), or supplementation of exogenous factors (Herrera‐Rincon et al., 2018; Murugan et al., 2022). Although these attempts successfully enhanced limb regeneration at least partly, it remains unclear why the limb regenerative ability is restricted in adult *Xenopus* frogs.

One key factor that is crucial for promoting limb regeneration in *X. laevis* is fibroblast growth factor 10 (FGF10). Multiple studies have suggested the importance of FGF10 in *X. laevis* limb regeneration (Lin et al., 2013; Yokoyama et al., 2000). Furthermore, previous studies have shown that administration of FGF10 in tadpole hindlimb buds at a late developmental stage (stage 56) after amputation can partially restore their regenerative capacity (Yokoyama et al., 2001). A recent study also provided a model in which cartilage tissue negatively affects *X. laevis* limb regeneration but FGF10 suppresses this effect (Aztekin et al., 2021). Taking these earlier reports into consideration, we hypothesized that analysis of the downstream signaling of FGF10 in *X. laevis* limb regeneration could provide important clues toward elucidating the details of the regeneration enhancing mechanisms and developing more sophisticated methods to induce complete limb regeneration in *X. laevis*, like in urodele amphibians.

In the present study, we aimed to identify specific cell populations that respond to FGF10 signaling at the limited regenerative stage (stage 56) in *Xenopus* tadpoles. By performing single‐cell RNA sequencing (scRNA‐seq) analysis of hindlimb bud blastemas after amputation and implantation of FGF10‐soaked beads, we explored cell clusters exhibiting differential gene expression profiles depending on FGF10 administration. Here we propose that functions of some cell populations are required for improving organ regenerative ability by FGF10 signaling. In addition, we also suggest that some genes reported to regulate *Xenopus* tadpole tail regeneration may function via FGF10 signaling and regulate limb regeneration. Furthermore, we report a unique cell population whose gene expression profile was strongly affected by FGF10 treatment, implying its unrevealed function in *X. laevis* limb regeneration. Our findings provide important clues toward understanding how FGF10 signaling enhances the regenerative capacity of *X. laevis*.

## 2 MATERIALS AND METHODS

### 2.1 Animals

*X. laevis* tadpoles were purchased from domestic breeders (Watanabe Zoshoku, Hyogo, Japan) and maintained in 0.2% salt water at 20°C. Animal experiments were carried out in accordance with the Guidelines for Proper Conduct of Animal Experiments of Science Council of Japan.

### 2.2 Limb amputation, bead implantation, and cell preparation

Limb amputation and bead implantation were performed essentially as described previously (Aztekin et al., 2021; Yokoyama et al., 2001). Affi‐gel blue gel beads #1537301 (BioRad) were washed overnight with 1× PBS (137 mM NaCl, 2.7 mM KCl, 10 mM Na_2_HPO_4_, 1.76 mM KH_2_PO_4_) supplemented with 0.1% bovine serum albumin (BSA; Nacalai Tesque) to remove antiseptics. The beads were soaked overnight in an equal volume of 1× PBS supplemented with 0.1% BSA and with/without 2 mg/ml recombinant human FGF10 (Abcam) at 4°C.

Tadpoles at stage 56 were anesthetized with 0.02% MS‐222 (Sigma‐Aldrich) and mounted on a wet paper towel. Left and right hindlimb buds were amputated with scissors between the presumptive knee and ankle level, and two to four FGF10‐soaked or PBS‐soaked beads were implanted underneath the amputation plane of the left or right hindlimb buds, respectively, using fine tweezers and tungsten needles. Implanted tadpoles were recovered from anesthesia and maintained for 5 days in 0.2% salt water at 20°C. Tadpoles that retained two or more implanted beads at both limbs were then selected for analysis. We sampled the generated blastemas from the left (implanted FGF10 beads) and right (implanted PBS beads) limbs of 23 tadpoles.

Cell dissociation and live cell sorting were performed essentially as described previously (Kato et al., 2021; Tsujioka et al., 2015) with several modifications. Samples were shredded with a razor and dispersed into single cells enzymatically by gentle shaking for 15 min at room temperature in dissociation solution (1× PBS supplemented with 100 U/ml DNase I [Roche] and 0.25 mg/ml Liberase TH Research Grade [Roche]). Cells were passed through a 55‐μm nylon mesh and washed with 1× PBS, spun down at 500*g* for 5 min, then resuspended in sorting buffer (1× PBS supplemented with 1 mM EDTA, 25 mM HEPES, and 1% BSA) supplemented with 1/200 volume of 7‐amino‐actinomycin D (7‐AAD) viability dye (BioLegend), and the live cells (7‐AAD negative) were sorted using FACS Aria SORP (BD Biosciences). Doublet or multiplet events were gated out using height and width parameters of forward scatter and side scatter.

### 2.3 Single‐cell RNA sequencing (scRNA‐seq)

scRNA‐seq library preparation and sequencing were performed according to the manufacturer's protocols (https://www.10xgenomics.com). Single‐cell suspensions of 16,000 cells (amount for generation of scRNA‐seq library of about 10,000 cells) in sorting buffer were used to generate scRNA‐Seq libraries with Chromium Next GEM Single Cell 3′ Reagent Kits v3.1 (10x Genomics). The product was amplified by polymerase chain reaction and sequenced using a Novaseq 6000 (Illumina). The scRNA‐seq data have been deposited in the DNA DataBank of Japan under accession code DRA012681.

Output data were processed using Cell Ranger version 4.0.0 to generate gene count matrices. For mapping and read counting, we used a genome sequence file generated by combining the *X. laevis* genome version 9.1 sequence (Xla.v91.repeatMasked.fa) and mitochondrial genome sequence of genome version 9.2 (XL9_2.fa), and a gene model file generated by combining the gene model for genome version 9.1 (XL_9.1_v1.8.3.2.primaryTranscripts.gff3) and mitochondrial gene annotation of the gene model for genome version 9.2 (XENLA_9.2_Xenbase.gff3). The genome sequence files and the gene model files were obtained from Xenbase (http://www.xenbase.org/, RRID:SCR_003280) (Fortriede et al., 2020). The generated count matrices were analyzed using the Seurat package v4.0.5 (Hao et al., 2021). Cells from empty droplets were identified using DropletUtils package v1.14.1 (Griffiths et al., 2018; Lun et al., 2019). Low‐quality or dying cells whose mitochondrial gene count exceeded 20% of the total gene count were excluded (Ilicic et al., 2016). Presumptive doublets were excluded using DoubletFinder v2.0.3 (McGinnis et al., 2019). Total number of reads, rate of reads mapped to the genome, detected cell numbers, mean and median UMI counts per cell, and mean and median of detected gene numbers per cell are presented in Table S1. The gene counts were normalized to total expression of each cell and scaled. Then, linear dimensional reduction was performed by principal component analysis. For clustering the cells, we used FindNeighbors and FindClusters function on Seurat using PC1‐PC100, and processed data were visualized using UMAP (McInnes et al., 2018). The cell‐cycle phase was inferred using the CellCycleScoring function on Seurat with the gene sets defined previously (Aztekin et al., 2019). Published scRNA‐seq data of regenerating hindlimbs (Aztekin et al., 2021) were also analyzed as described above, and projected to our data to detect the corresponding cell clusters using FindTransferAnchors and MapQuery function on Seurat. Annotation of cell types was basically performed by comparing the expression profiles of marker genes used in a previous report (Aztekin et al., 2021). The names of annotated cell clusters followed the previous report for easier comparison between their data and ours. Clusters that could not be identified unambiguously were assigned a label with a suffix of “‐like” or with a symbol of a gene expressed characteristically in the cluster.

Differentially expressed genes (DEGs) were detected using the FindMarker function on Seurat with an adjusted *p*‐value threshold <0.05. Enrichment analysis was performed on Metascape (http://metascape.org) (Zhou et al., 2019). As background genes in the analysis, we used 14,249 *M. musculus* genes that have *X. laevis* orthologs (*E*‐values <1 × 10^−10^).

## 3 RESULTS

### 3.1 scRNA‐seq on FGF10‐treated blastemas successfully captured the effect of FGF10 treatment on gene expression profiles

**FIGURE 1:**
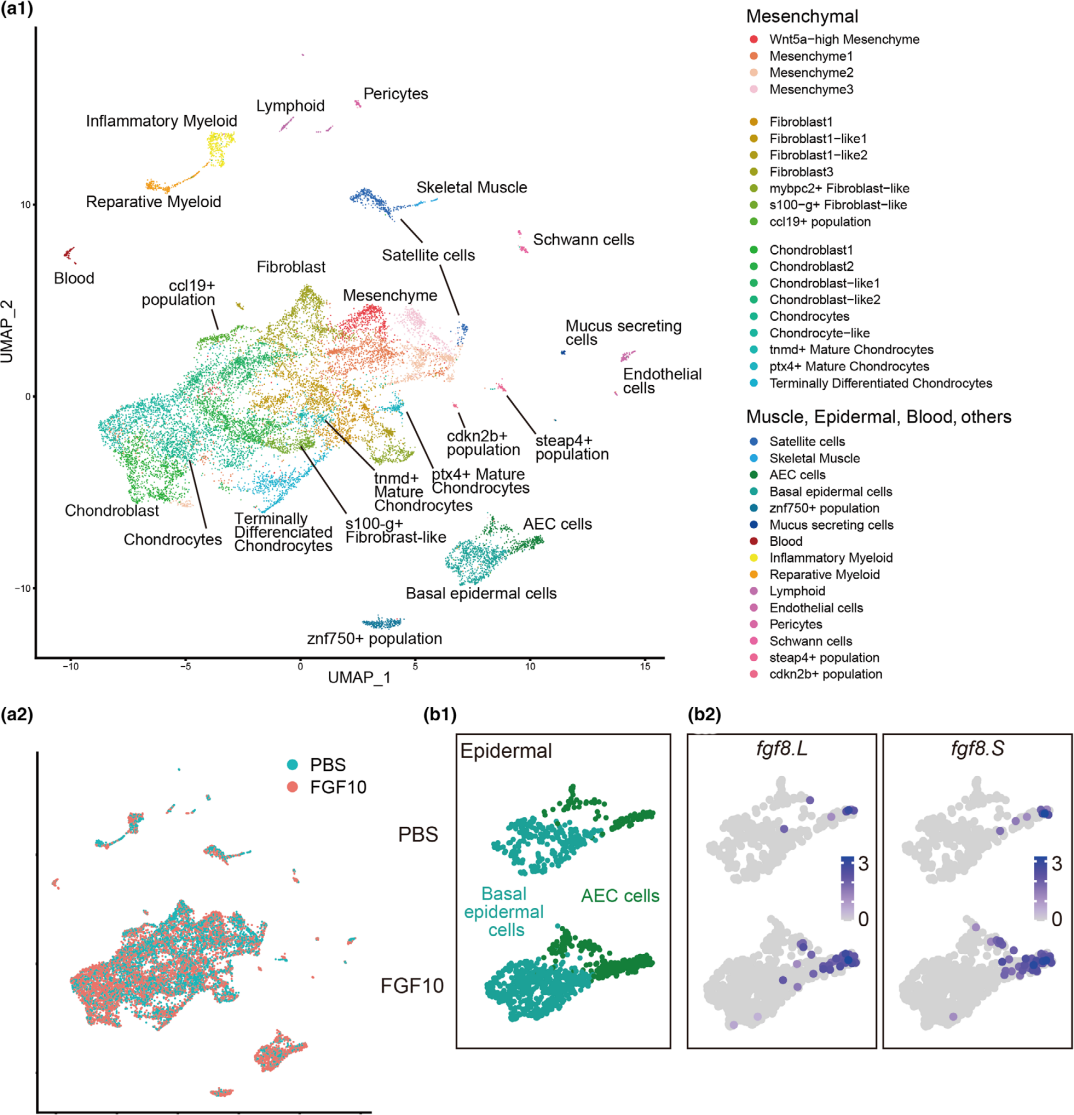
Single‐cell transcriptomics on the FGF10‐treated and PBS‐treated hindlimb blastemas. (a1‐2) UMAP plot of cells detected in the scRNA‐seq. (a1) cells from the FGF10‐treated and PBS‐treated samples were combined and divided into 35 clusters depending on the similarity of the gene expression profiles in each cell. Colors indicate cluster identity. Expression profiles of several marker genes in each cluster are shown in Figure S2. (a2) UMAP plot with colors indicating sample condition. (b1‐2) The scRNA‐seq data showed expansion of the AEC cluster and *fgf8*‐expressing cells in the FGF10‐treated sample. (b1) UMAP plot of the AEC and basal epidermal cell clusters of the PBS‐treated (top) and FGF10‐treated (bottom) samples. (b2) Normalized expression levels (transcripts per 10^4^) of *fgf8.L* and *fgf8.S* in the epidermal clusters of each cell of the PBS‐treated (top) and FGF10‐treated (bottom) samples are plotted in natural‐log transformed scale shown by color depth of blue

With the aim to analyze the effect of FGF10 treatment on gene expression profiles of amputated *Xenopus* tadpole hindlimbs at the single‐cell level, we performed scRNA‐seq analysis. For this, hindlimbs of stage‐56 tadpoles were amputated, and FGF10‐soaked and control PBS‐soaked beads were implanted into the left and right amputated limbs of each tadpole, respectively (Figure S1). At 5 days after amputation when blastemas covered by wound epidermis had been generated (Beck et al., 2009), blastemas were dissected and dispersed enzymatically into single cells, followed by scRNA‐seq. We detected 8,266 and 7,540 cells in the FGF10‐treated and control PBS‐treated samples, respectively. Cells were classified into 35 clusters, and presumptive cell types were annotated (Figure 1a) on the basis of their expression profiles of various marker genes (Aztekin et al., 2021) (Figure S2). We detected clusters corresponding to epidermal, mesenchymal, muscle, blood, and endothelial lineages, suggesting that our data cover a broad range of tissues in the regenerating hindlimbs. To confirm that the FGF10 treatment by bead implantation was successful, we checked the expression of genes regulated by FGF10. The data showed that *fgf8*‐expression in the apical epidermal cap (AEC) was expanded by FGF10 treatment (Figures 1b and S3a), consistent with a previous report (Yokoyama et al., 2001). In addition, the data showed that FGF10 treatment significantly upregulated *wnt3a.L* expression in the AEC (Figure S3b); during limb development, *fgf10* induces *wnt3a* and *fgf8* in the apical epidermal ridge (AER) and *fgf8* expression is stabilized by *wnt3a* (Kawakami et al., 2001; Kengaku et al., 1998). These results suggested that the FGF10 treatment was successful and our data well captured the effect of FGF10 treatment on the gene expression profiles of the blastemas.

### 3.2 Overview of the transcriptomics data

Among the 35 clusters, the cell numbers in several clusters differed between the FGF10‐treated and PBS‐treated samples (Table 1). The epidermal clusters (AEC cells, basal epidermal cells, and zinc finger protein 750 [znf750+] population), the chondrocyte cluster, and the chondrocyte‐like cluster had much larger numbers of cells in the FGF10‐treated sample than in the PBS‐treated sample, whereas the number of cells in the tenomodulin+ (tnmd+) mature chondrocytes, the mesenchyme3, the fibroblast3, and the chondroblast‐like1 clusters were smaller in the FGF10‐treated sample than in the PBS‐treated sample. Although the transcriptomics data have no replicates and thus the differences in the cell numbers could not be confirmed statistically, it is possible that FGF10 treatment mainly affected these clusters; indeed, expansion of the epidermal clusters may be supported by the upregulation of *fgf8* and *wnt3a* (Figures 1b and S3).

**TABLE 1:** Numbers of cells and detected DEGs in each cluster.

Cluster annotation	Number of cells		Upregulated DEGs				Downregulated DEGs			
	PBS	FGF10	All	RPs^a^	Other than RPs^a^	Fc^b^ >2	All	RPs^a^	Other than RPs^a^	Fc^b^ <0.5
Wnt5a‐high mesenchyme	256	340	0	0	0	0	0	0	0	0
Mesenchyme1	598	468	6	1	5	0	21	2	19	0
Mesenchyme2	339	236	5	0	5	3	83	0	83	1
Mesenchyme3	289	132	1	0	1	1	0	0	0	0
Fibroblast1	361	323	28	5	23	0	29	4	25	1
Fibroblast1‐like1	365	495	9	0	9	1	8	8	0	0
Fibroblast1‐like2	186	228	1	0	1	0	2	1	1	0
Fibroblast3	560	308	5	0	5	0	25	5	20	0
mybpc2+ fibroblast‐like	175	99	2	2	0	0	2	0	2	0
s100‐g+ fibroblast‐like	248	203	27	20	^c^(7)	0	66	1	65	3
ccl19+ population	127	138	3	0	3	0	6	2	4	0
Chondroblast1	325	666	6	0	6	0	38	0	38	0
Chondroblast2	459	503	3	0	3	0	10	5	5	0
Chondroblast‐like1	486	293	1	0	1	0	7	4	3	0
Chondroblast‐like2	195	172	0	0	0	0	6	5	1	1
Chondrocytes	490	689	9	0	9	0	36	0	36	1
Chondrocyte‐like	306	603	7	2	5	0	22	0	22	0
tnmd+ mature chondrocytes	172	49	0	0	0	0	2	0	2	1
ptx4+ mature chondrocytes	91	66	0	0	0	0	1	1	0	0
Term. diff. chondrocytes^d^	277	309	2	0	2	0	3	2	1	0
Satellite cells	207	204	3	0	3	0	4	2	2	0
Skeletal muscle	30	30	0	0	0	0	0	0	0	0
AEC cells	91	291	5	0	5	5	3	0	3	0
Basal epidermal cells	259	598	13	0	13	5	16	0	16	0
znf750+ population	74	202	1	1	0	0	1	0	1	0
Mucus‐secreting cells	21	29	0	0	0	0	0	0	0	0
Blood	29	68	0	0	0	0	0	0	0	0
Inflammatory myeloid	166	188	0	0	0	0	0	0	0	0
Reparative myeloid	113	103	10	0	10	8	0	0	0	0
Lymphoid	68	48	0	0	0	0	0	0	0	0
Endothelial cells	54	62	0	0	0	0	0	0	0	0
Pericytes	40	26	0	0	0	0	0	0	0	0
Glial cells	44	60	0	0	0	0	0	0	0	0
steap4+ population	30	15	2	0	2	2	85	73	(12)^c^	0
cdkn2b+ population	9	22	0	0	0	0	0	0	0	0

^a^ RPs, ribosomal protein genes.

^b^ Fc, fold change of gene expression level.

^c^ Excluded from the analysis.

^d^ Term. diff. chondrocytes, terminally differentiated chondrocytes.

As FGF10 treatment enhances regenerative ability, we checked the nature of cells in the proliferative state in each cluster. We inferred the cell‐cycle phase on the basis of the expression of cell‐cycle‐phase‐associated genes, and compared the ratio of numbers of cells at the G1 and S/G2/M phases in each cluster of the FGF10‐treated and PBS‐treated samples (Table 2). We found that the satellite cell cluster, a cluster of muscle stem cells with high expression of *paired box 7.L* (*pax7.L*) (Figure S2) (Seale et al., 2000), had higher proliferation in the FGF10‐treated blastemas than in the PBS‐treated blastemas (Table 2), probably reflecting the facilitated muscle regeneration by FGF10 treatment. In contrast, the terminally differentiated chondrocyte cluster exhibited lower proliferation in the FGF10‐treated blastemas (Table 2), maybe reflecting the effect of FGF10 treatment on chondrogenesis, consistent with a previous report that FGF10 treatment inhibits chondrogenesis in limb explants (Aztekin et al., 2021).

**TABLE 2:** Numbers of cells with inferred cell‐cycle phase in each cluster.

Cluster annotation	PBS		FGF10		Ratio of S/G2/M^a^
	G1	S/G2/M	G1	S/G2/M	
Wnt5a‐high mesenchyme	119	137	200	140	Low*
Mesenchyme1	315	283	232	236	
Mesenchyme2	0	339	0	236	
Mesenchyme3	67	222	50	82	Low*
Fibroblast1	308	53	281	42	
Fibroblast1‐like1	297	68	389	106	
Fibroblast1‐like2	165	21	188	40	
Fibroblast3	474	86	259	49	
mybpc2+ fibroblast‐like	158	17	90	9	
s100‐g+ fibroblast‐like	196	52	177	26	Low*
ccl19+ population	123	4	132	6	
Chondroblast1	218	107	476	190	
Chondroblast2	388	71	419	84	
Chondroblast‐like1	452	34	260	33	High*
Chondroblast‐like2	189	6	161	11	
Chondrocytes	338	152	478	211	
Chondrocyte‐like	290	16	570	33	
tnmd+ mature chondrocytes	140	32	36	13	
ptx4+ mature chondrocytes	64	27	45	21	
Terminally differentiated chondrocytes	218	59	269	40	Low*
Satellite cells	99	108	76	128	High*
Skeletal muscle	27	3	22	8	
AEC cells	33	58	128	163	
Basal epidermal cells	157	102	403	195	
znf750+ population	54	20	142	60	
Mucus‐secreting cells	14	7	23	6	
Blood	26	3	45	23	High*
Inflammatory myeloid	138	28	152	36	
Reparative myeloid	108	5	98	5	
Lymphoid	41	27	28	20	
Endothelial cells	41	13	40	22	
Pericytes	33	7	23	3	
Glial cells	28	16	45	15	
steap4+ population	28	2	11	4	
cdkn2b+ population	9	0	21	1	
Sum	5,355	2,185	5,969	2,297	

^a^ Statistical significance of difference in the ratio of cells at the G1 phase and S/G2/M phase in each cluster of the PBS‐treated or FGF10‐treated samples was tested using Fisher's exact test. *p < 0.05. “High” or “Low” means that the cluster contains higher or lower ratio of cells at S/G2/M phase in the FGF10‐treated sample than in the PBS‐treated sample. Blank cells mean that there were no significant differences.

### 3.3 Gene expression profiles at the whole transcriptome level reflected the expanded epidermal clusters and inhibited chondrogenesis in the FGF10‐treated blastemas

We investigated the effect of FGF10 treatment on the gene expression profiles of the blastemas at the whole transcriptome level. The ten genes with the largest fold change in expression among the upregulated genes in the FGF10‐treated blastemas contained keratins *(keratin 12.L*, *keratin 12.S*, *Xelaev18016080m.g*), hemoglobins (*hemoglobin subunit gamma 2.L*, *hemoglobin subunit delta.S*, *Xelaev18045099m.g*, *Xelaev18045098m.g*), cystatins (*loc100049121.S*, *loc101734526.L*), and *cytokine‐like 1* (*cytl1.L*) (Table S2). Detection of these genes as upregulated genes at the whole transcriptome level was probably due to the expansion of expressing cells in the FGF10‐treated blastemas because the average expression level of these genes in each cluster was not drastically different between the FGF10‐treated and PBS‐treated blastemas (Figure S4). Keratins, a family of structural fibrous proteins expressed in epithelial cells, and cystatins, a family of cysteine protease inhibitors, were specifically expressed in the epidermal clusters (basal epidermal cells, znf750+ population; Figure S4), reflecting expansion of the epidermal clusters in the FGF10‐treated blastemas (Table 1). The hemoglobins expressed in the blood cell cluster (Figure S4) were detected as upregulated genes in the FGF10‐treated blastemas, maybe due to the larger number of blood cells contained in the FGF10‐treated sample (Table 1). *cytl1.L*, whose mouse ortholog is reported to be required for the maintenance of cartilage homeostasis (Jeon et al., 2011), was expressed in the chondrocyte‐like cluster (Figure S4). The fact that *cytl1.L* was detected as an upregulated gene may be due to the larger yield cells of the chondrocyte‐like clusters in the FGF10‐treated sample (Table 1).

On the other hand, the downregulated genes in the FGF10‐treated blastemas contained genes associated with chondrogenesis; the ten genes that showed the largest fold change in expression among the downregulated genes in the FGF10‐treated blastemas contained collagens (*collagen type III alpha 1* and *collagen type XII alpha 1*; *col3a1.L*, *col12a1.L*), *versican.L* (*vcan.L*), and *zinc finger homeodomain 4.S* (*zfhx4.S*) (Table S3 and Figure S4). Collagen is a main structural protein in the extracellular matrix found in connective tissue such as cartilage (Alcaide‐Ruggiero et al., 2021). *vcan* is reported to facilitate chondrogenesis in mice (Choocheep et al., 2010). *zfhx4* is highly expressed in cartilage and coordinates the endochondral ossification in mice (Nakamura et al., 2021). The downregulation of these genes probably reflects the FGF10‐mediated inhibition of chondrogenesis (Aztekin et al., 2021).

### 3.4 Analysis at the cluster level of identified cell types that respond to FGF10 treatment

To investigate the effect of FGF10 treatment on the gene expression profiles of each cluster, we searched for cell clusters whose gene expression profiles were affected by the FGF10 treatment. We screened differentially expressed genes (DEGs) between each corresponding cluster of the FGF10‐treated and PBS‐treated samples, and detected DEGs in several clusters (Table 1) (hereafter, DEGs that were upregulated or downregulated in the FGF10‐treated sample are simply referred to as “upregulated DEGs” or “downregulated DEGs,” respectively). Although ribosomal protein genes were detected as DEGs in some clusters, they were excluded from the analysis, because ribosomal protein genes are housekeeping genes. In addition, because many ribosomal protein genes were detected as DEGs in both the “*S100 calcium‐binding protein G*+ (s100‐g+) fibroblast‐like” and “*six transmembrane epithelial antigen of prostate 4+* (steap4+) population” clusters, we could not rule out the possibility that normalization of gene expression levels in these clusters was biased toward high (s100‐g+ fibroblast‐like) or low (steap4+ population) in the FGF10‐treated samples, respectively. Therefore, we also excluded the upregulated DEGs in the s100‐g+ fibroblast‐like cluster and downregulated DEGs in the steap4+ population cluster from the analysis.

**FIGURE 2:**
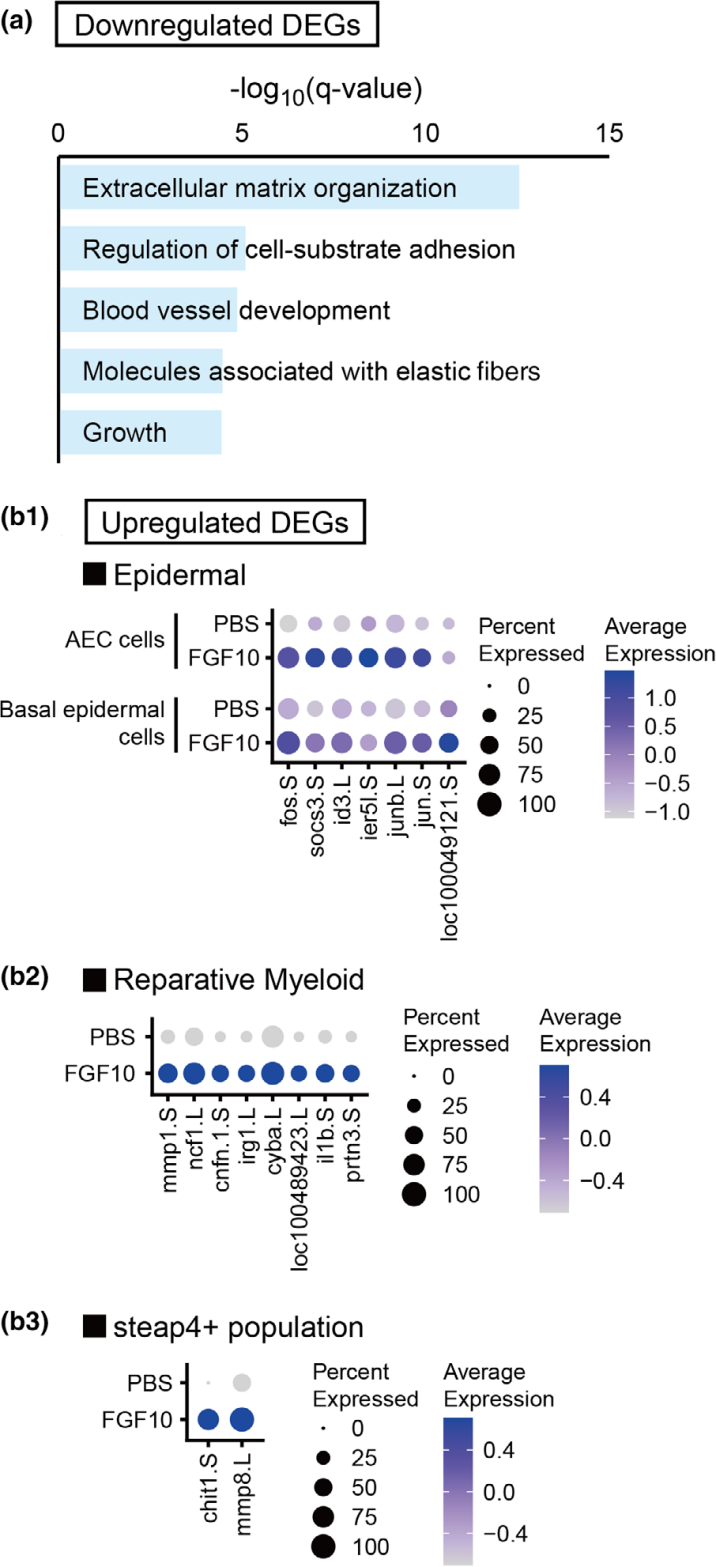
Effect of FGF10 treatment on gene expression profiles of each cluster. (a) Mouse orthologs of all the downregulated DEGs (Fc <0.5) detected in each cluster were searched, and the identified mouse orthologs were subjected to enrichment analysis. The terms with the five lowest *q*‐values in the enrichment analysis are shown. (b1–3) Comparison of the expression levels of upregulated DEGs (Fc >2) detected in the epidermal (b1), reparative myeloid (b2), and steap4+ population (b3) clusters between PBS‐treated and FGF10‐treated conditions. Average expression level is shown as *z*‐score (quotient of the difference between the mean expression value of cells in the cluster and the mean expression value of all cells, and the standard deviation of expression of all cells) calculated with the mean expression value of cells in indicated clusters of each condition (PBS or FGF10) and of both conditions, indicated by dot color depth. Percentage of expressing cells in each cluster is indicated by dot size. Mouse orthologs of *loc100049121.S* in (b1) and *loc100489423.L* in (b2) are *cystatin B* (*cstb*) and *toll‐like receptor 5* (*tlr5*), respectively

The majority of the detected DEGs were downregulated DEGs (349 of 460 DEGs). Enrichment analysis revealed that genes associated with the term “extracellular matrix organization” were enriched among the downregulated DEGs (Figure 2a), and the downregulated DEGs contributing to the term contained genes coding for several types of collagens (Table S4) such as *col3a1.L* and *col12a1.L*, which were detected as downregulated genes at the whole transcriptome level as mentioned above. These results seem consistent with FGF10‐mediated inhibition of chondrogenesis, a suggested mechanism of the FGF10‐mediated improvement of regenerative capacity; some regeneration‐inhibitory factors are secreted during chondrogenesis, and FGF10 suppresses chondrogenesis (Aztekin et al., 2021). The mesenchyme2 and s100‐g+ fibroblast‐like clusters contained relatively large numbers of downregulated DEGs (Table 1), and genes associated with the term “collagen chain trimerization” were enriched among these DEGs (Figure S5), including *col3a1.L* and *col12a1.L*. The s100‐g+ fibroblast‐like cluster in the FGF10‐treated blastemas exhibited a lower proliferative state (Table 2). These findings suggest that FGF10 especially affected these clusters, although expression of *fgf receptors* in the 2 clusters was not particularly high (Figure S6).

The fibroblast1 cluster had a relatively large number of both upregulated and downregulated DEGs (Table 1), suggesting that this cluster is also affected by FGF10‐treatment. The downregulated DEGs in the fibroblast1 cluster (Figure S7) contained *vcan.L* and *col12a1.L*, which were also detected as downregulated genes at the whole transcriptome level, suggesting inhibited chondrogenesis. We also found that the upregulated DEGs contained *short stature homeobox 2.L* (*shox2.L*) (Figure S7), a proximal marker gene in mouse limb development (Yu et al., 2007). Therefore it is possible that the proximal nature was expanded by FGF10 treatment.

On the other hand, although the number of upregulated DEGs was rather small among all the DEGs, many of them showed a high fold change in the expression level (Fc) between corresponding clusters of the FGF10‐treated and PBS‐treated samples; 25 of the 111 upregulated DEGs had an Fc >2, whereas the downregulated DEGs with an Fc <0.5 accounted for only 8 of 349 (Table 1 and Figure 2b). Many of the upregulated DEGs with a high Fc were detected in the AEC, basal epidermal cell, and reparative myeloid clusters (Figure 2b).

The importance of the epidermis in organ regeneration, particularly the specialized wound epidermis or AEC that is formed in response to injury or amputation, has been reported in many experimental systems or species (Han et al., 2001; Mescher, 1976; Miyazaki et al., 1996; Pearl et al., 2008; Thornton, 1957), and its importance in the FGF10‐mediated improvement of limb regenerative capacity has also been reported (Aztekin et al., 2021; Yokoyama et al., 2001). The upregulated DEGs of the AEC and basal epidermal cells (Figure 2b1 and Table S5) contained components of activator protein 1 (AP‐1) transcription factor complex, *jun.S*, *junb.L*, and *fos.S*. *junb* is required for *Xenopus* tadpole tail regeneration (Nakamura et al., 2020). Our findings suggest an important role of AP‐1 signaling in the epidermis for the FGF10‐mediated improvement of limb regenerative ability.

The reparative myeloid cluster that is inferred to correspond to the “reparative myeloid” of tadpoles, suggested to be required for tail regeneration (Aztekin et al., 2020), also showed a remarkably altered gene expression profile (Figure 2b2 and Table S5). The upregulated DEGs in the reparative myeloid contained *cytochrome b‐245 α polypeptide.L* (*cyba.L*). In *Xenopus* tadpoles, reactive oxygen species (ROS) are required for successful tail regeneration, as tail regeneration is impaired by knockdown of *cyba*, a subunit of NADPH oxidase that mediates ROS production (Love et al., 2013). Thus, our result also suggests that an ROS‐mediated pathway in the reparative myeloid has an important role in the improvement of regenerative ability by FGF10 administration.

**FIGURE 3:**
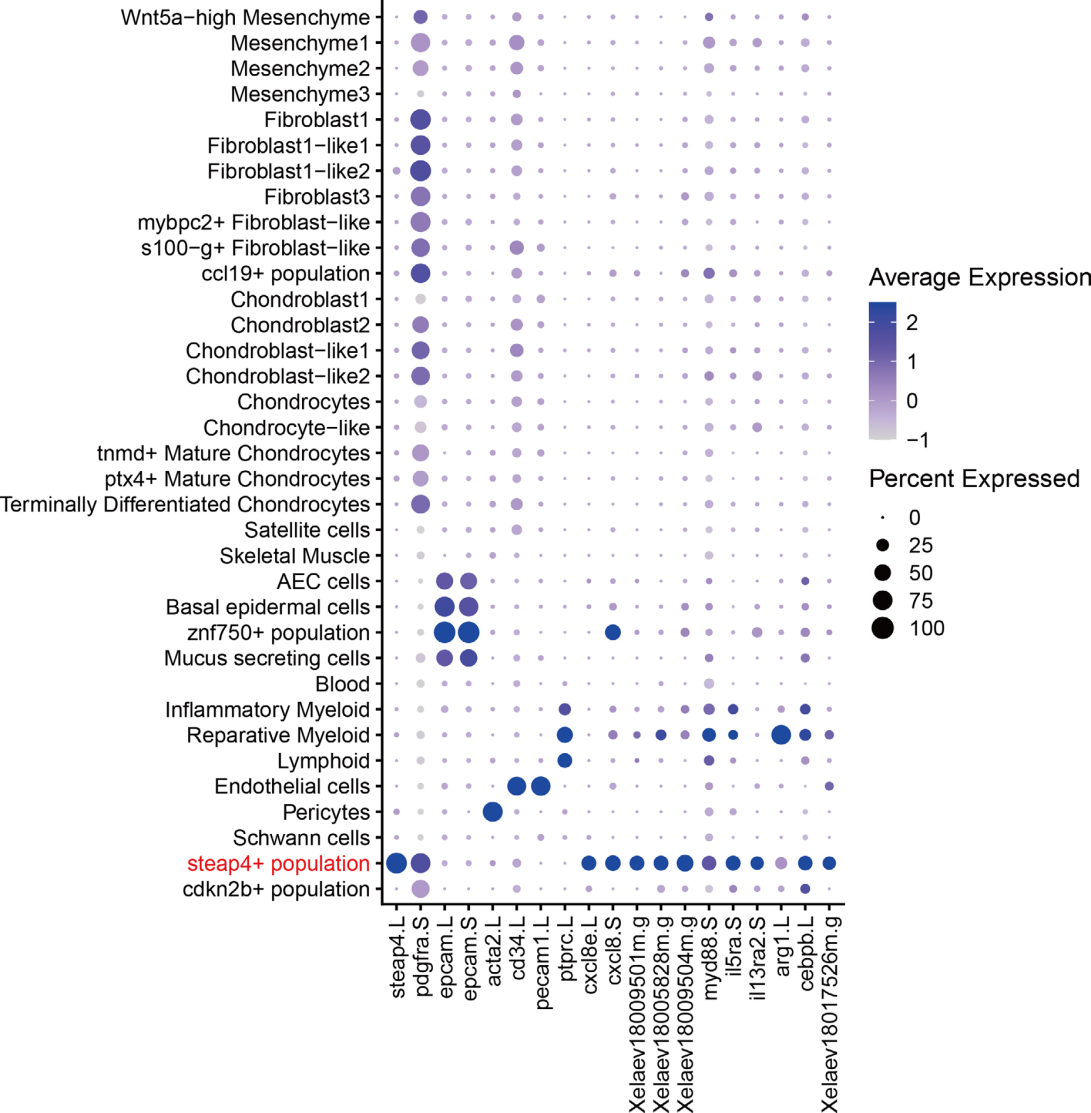
Characterization of the steap4+ population. Expression of several lineage marker genes across all clusters is shown. Average expression level is shown as *z*‐score calculated using the mean expression value of the indicated cluster in both conditions (PBS and FGF10) and of all cells, indicated by dot color depth. Percentage of expressing cells in each cluster is indicated by dot size. This cluster hardly/weakly expressed epidermal (*epcam.L/S*), endothelial (*cd34.L*, *pecam1.L*), myofibroblast (*acta2.L*), and leukocyte (*ptprc.L*) markers, and expressed a fibroblastic gene (*pdgfra.S*), chemokines (*cxcl8e.L*, *cxcl8.S*, *Xelaev18009501m.G* [*cxcl1*], *Xelaev18005828m.G* [*cxcl5*], *Xelaev18009504m.G* [*cxcl5*]), and immune‐related genes (*myd88.S*, *il5ra.S*, *il13ra2.S*, *arg1.L*, *cebpb.L*, *Xelaev18017526m.G* [*irak3*]). Mouse orthologs of each unnamed gene are shown in square brackets

We also noticed that two upregulated DEGs, *mmp8.L* and *chit1.S*, in the cluster of the steap4+ population, showed the highest Fc (*mmp8.L*: Fc = 17.0, *chit1.S*: Fc = 13.5) among all upregulated DEGs (Figure 2b3 and Table S5). The steap4+ population exclusively expressed *steap4* (Figure 3), which encodes a metalloreductase involved in the transport of copper and iron and is expressed in adipose tissue, hepatocytes, and pancreatic islets/ β‐cells; its expression is modulated in response to inflammation (Scarl et al., 2017), but there are no reports linking *steap4* function to regeneration. *mmp8.L* encodes matrix metalloproteinase‐8, whose mammalian homolog is expressed in neutrophils, chondrocytes, and rheumatoid synovial fibroblasts (Cole et al., 1996; Hanemaaijer et al., 1997; Hasty et al., 1990). *chit1.S* encodes chitinase1, which catalyzes the degradation of chitin. Chitinase plays an important role in the biological defense against pathogens containing chitin, such as fungus (Chang et al., 2020). In mammals, *chit1* is expressed in activated macrophages, neutrophils, and epithelial cells (Kanneganti et al., 2012; van Eijk et al., 2005). Although direct evidence for the requirement of these genes in organ regeneration is lacking, their abundant expression in the specific cell population of the FGF10‐treated sample raises the possibility that the steap4+ population is involved in the FGF10‐mediated improvement of limb regenerative ability.

To investigate whether the steap4+ population is observed in the regenerating limbs of tadpoles at an earlier stage when limb buds exhibit complete regeneration, the scRNA‐seq data of regenerating hindlimb buds of stage 52 tadpoles reported previously (Aztekin et al., 2021) were analyzed, but the corresponding cell population was not detected (Figure S8 and Table S7). The steap4+ population was not detected even in data of regenerating hindlimbs of stage 56 tadpoles, which corresponds to our PBS‐treated sample (Figure S8 and Table S7). The steap4+ population showed unique gene expression profiles among the clusters; the steap4+ population expressed several immune‐related genes and hardly/weakly expressed epidermal, endothelial, myofibroblast, and leukocyte markers, but the cell type of the steap4+ population cannot be annotated unambiguously, although the cell type could be annotated as a fibroblast‐like cluster on the basis of their *platelet‐derived growth factor receptor alpha.L* (*pdgfra.L*) expression (Figure 3). Further analysis is needed to characterize this newly identified cell population and its involvement in the regenerative capacity.

## 4 DISCUSSION

In the present study, we performed single‐cell transcriptomics of FGF10‐treated regenerating hindlimbs to analyze the FGF10‐mediated improvement of regenerative capacity. We observed expansion of the AEC cell cluster with *fgf8* and *wnt3a* expression (Figures 1b and S3), and signatures of inhibited chondrogenesis (Figurea 2a, S4, and S5) in the FGF10‐treatment blastemas, consistent with previous reports (Aztekin et al., 2021; Kawakami et al., 2001; Kengaku et al., 1998; Yokoyama et al., 2001). We also observed significant upregulation of *wnt5a.S* and *wnt5b.L* in the FGF10‐treated blastemas (Figure S3). Previous studies reported the involvement of *wnt5a* and *wnt5b* in limb regeneration (Ghosh et al., 2008), and the functions of *wnt5a* during developmental/regenerative elongation/outgrowth of body parts (Andre et al., 2015; Kilian et al., 2003; Sugiura et al., 2009; Yamaguchi et al., 1999). Our results further support the requirement of these wnts for FGF10‐mediated improvement of regenerative capacity.

scRNA‐seq was performed with cells from enzymatically dispersed tissues, and the yields of the cell types were affected by the tissue dispersion processes. A high yield of some tissue types does not always indicate that the sample contains a high amount of the tissue type; the dispersibility of the tissue affects the yield. The scRNA‐seq data showed larger numbers of cells of the chondrocytes and chondrocyte‐like clusters in the FGF10‐treated blastemas than in the PBS‐treated blastemas (Table 1). Some possible explanations for this are as follows: (1) FGF10‐treated blastemas contained larger amounts of cells of these clusters, and (2) the cells of these clusters in the FGF10‐treated blastemas were sensitive to enzymatic dispersion for some reason, for example, downregulation of several extracellular matrix components such as collagens. These possibilities should be investigated in future studies.

We analyzed the proliferative state of each detected cluster (Table 2). We observed a lower proliferative state in the Wnt5a‐high mesenchyme and mesenchyme3 clusters (Table 2). It is possible that these clusters contained precursors of a chondrogenic lineage and were affected by FGF10. We also found, however, that the chondroblast‐like1 cluster exhibited a higher proliferative state in the FGF10‐treated blastemas (Table 2). Among the chondroblastic clusters (chondroblast1, chondroblast2, chondroblast‐like1, and chondroblast‐like2), the chondroblast‐like1 cluster had a less chondroblastic character; the cluster had weaker expression of *sox9*, a chondrogenic marker (Lefebvre & Dvir‐Ginzberg, 2017). This cluster may have different features and a different responsiveness compared with other chondroblastic clusters. We also found that the blood cluster in the FGF10‐treated blastemas was in a higher proliferative state (Table 2), that is, the expression of cell‐cycle‐associated genes was detected, although there are no reports, to our knowledge, describing the cell cycle of *Xenopus* nucleated erythrocytes except that RNA synthesis is detected therein (Maclean et al., 1973). In newt, erythrocytes are reported to be capable of delivering multiple secretory factors, including growth factors and matrix metalloproteases, to the blastema (Casco‐Robles et al., 2018). The cell number of the blood cluster was larger in the FGF10‐treated blastemas than in the PBS‐treated blastemas (Table 1), suggesting the possible involvement of erythrocytes in FGF10‐mediated limb regeneration, although we cannot rule out the possibility that these findings were an artifact.

We compared gene expression profiles of both samples at the whole transcriptome level, and detected *gap junction protein alpha 1.L* (*gja1.L*) as a downregulated gene in the FGF10‐treated sample (Table S3). *gja1* encodes connexin 43, a component of a gap junction channel (Söhl & Willecke, 2004) that is required for the appropriate expression of morphogens and thus limb bud patterning (Allen et al., 1990; Makarenkova & Patel, 1999; Söhl & Willecke, 2004). Downregulation of *gja1.L* in the FGF10‐treated sample might suggest that precise control of its expression is required for limb morphogenesis. We also detected *twist family bHLH transcription factor 1.L* (*twist1.L*) as a downregulated gene in the FGF10‐treated sample (Table S3).This finding, however, seems somehow paradoxical, considering that *twist1* is induced by Wnt3a and inhibits chondrogenesis in a chondrogenic culture cell line (Reinhold et al., 2006). Our findings indicates that FGF10 treatment upregulated *wnt3a.L* expression (Figure S3) and impaired chondrogenesis (Figure 2a; Figures S4 and S5). It might be that the FGF10‐mediated inhibition of chondrogenesis is elicited by interfering with the Wnt3a‐twist1 pathway, or via another pathway that downregulates *twist1*. *cathepsin H.L* (*ctsh.L*) and *granzyme H.L* (*gzmh.L*), which were expressed in the leukocytes cluster (Figure S4) and were also detected as downregulated genes in the FGF10‐treated blastemas (Table S3). Cathepsins and granzymes are proteases involved in antigen presentation and cell‐mediated cytotoxicity in the acquired immune system, respectively (Nanut et al., 2014). We and other groups previously reported regeneration‐inhibitory aspects of the immune response (Aztekin et al., 2020; Fukazawa et al., 2009; King et al., 2012); the downregulation of these genes in the FGF10‐treated blastemas provides support that appropriate regulation of immune responses is also required for limb regeneration. *xa‐1‐like.S* and *Xelaev18003895m.g* were also detected as downregulated genes in the FGF10‐treated blastemas (Table S3), although their orthologs in other species were not detected and thus it is difficult to guess their functions; their significant downregulation suggests their possible involvement in the loss of regenerative capacity that can be improved by FGF10.

Analysis at the cluster level identified cell types that respond to FGF10 treatment. Some differences exist in the range of detected cell types between our scRNA‐seq data and the data of regenerating hindlimb buds reported previously (Aztekin et al., 2021) (Table S7). In particular, the steap4+ population was not detected in the previous data (Figure S8), a difference that might be due to differences in the tissue dispersion protocols used. The previous report used trypsin, whereas we used a cocktail of collagenase I, collagenase II, and thermolysin, for tissue dispersion, which probably elicited a different dispersion spectrum of tissue types. It is also possible that other experimental conditions such as the animal strain used and the experimental temperature led to differences in the yields of various cell types.

In this study, we detected drastic differences in the gene expression profiles of a subset of epidermal cells and myeloid cells, as well as in newly identified steap4+ population in the FGF10‐treated sample, suggesting the involvement of these clusters in the FGF10‐mediated improvement of limb regenerative ability. Our study provides fundamental data of cell populations whose gene expression profiles correlate with the regenerative capacity and insights into the molecular and cellular bases underlying the FGF10‐mediated improvements of regenerative capacity.

## Supporting information


**Figure S1.** Morphologies of the PBS‐treated and FGF10‐treated blastemas.
**Figure S2.** Expression profiles of various lineage marker genes and characteristic genes in mesenchymal and muscle (a), epidermal, blood, endothelial, and other (b) clusters.
**Figure S3.** FGF10‐treatment upregulated its downstream genes.
**Figure S4.** The top 10 upregulated and downregulated genes in the FGF10‐treated blastemas at the whole transcriptome level.
**Figure S5.** Enrichment analysis using mouse orthologues of downregulated DEGs detected in the Mesenchyme2 (a) and s100‐g+ Fibroblast‐like (b) clusters.
**Figure S6.** Expression of Fgf receptor genes in each cluster.
**Figure S7.** Comparison of the expression levels of upregulated and downregulated DEGs detected in the Fibroblast1 cluster.
**Figure S8.** The scRNA‐seq data of intact hindlimbs at st. 52 and 56, and blastemas of 5 dpa regenerating hindlimbs in tadpoles at st. 52, 56, and 58 reported previously (Aztekin et al., 2021) were projected to our scRNA‐seq data to detect their corresponding clusters.
**Table S1.** Summary and quality control of the scRNA‐seq data.
**Table S2.** Genes that were upregulated in the FGF10‐treated blastemas at the whole transcriptome level. The top 10 upregulated genes are shown.
**Table S3.** Genes that were downregulated in the FGF10‐treated blastemas at the whole transcriptome level. The top 10 downregulated genes are shown.
**Table S4.** Downregulated DEGs contributing to each term in the enrichment analysis.
**Table S5.** Upregulated DEGs with Fc >2 and their expression profiles.
**Table S6.** Downregulated DEGs with Fc <0.5 and their expression profiles.
**Table S7.** Numbers of cells in the previous report (Aztekin et al., 2021) that were projected to our data for annotation of corresponding clusters.
