# Supplementary material for: Cellular responses in the FGF10‐mediated improvement of hindlimb regenerative capacity in Xenopus laevis revealed by single‐cell transcriptomics
Source: Dev Growth Differ. 2022 Jun 20;64(6):266–78. doi: 10.1111/dgd.12795 (PMC11520959; doi:10.1111/dgd.12795)
Supplement: Supplementary file 1 — Figure S1. Morphologies of the PBS‐treated and FGF10‐treated blastemas. Figure S2. Expression profiles of various lineage marker genes and characteristic genes in mesenchymal and muscle (a), epidermal, blood, endothelial, and other (b) clusters. Figure S3. FGF10‐treatment upregulated its downstream genes. Figure S4. The top 10 upregulated and downregulated genes in the FGF10‐treated blastemas at the whole transcriptome level. Figure S5. Enrichment analysis using mouse orthologues of downregulated DEGs detected in the Mesenchyme2 (a) and s100‐g+ Fibroblast‐like (b) clusters. Figure S6. Expression of Fgf receptor genes in each cluster. Figure S7. Comparison of the expression levels of upregulated and downregulated DEGs detected in the Fibroblast1 cluster. Figure S8. The scRNA‐seq data of intact hindlimbs at st. 52 and 56, and blastemas of 5 dpa regenerating hindlimbs in tadpoles at st. 52, 56, and 58 reported previously (Aztekin et al., 2021) were projected to our scRNA‐seq data to detect their corresponding clusters. Table S1. Summary and quality control of the scRNA‐seq data. Table S2. Genes that were upregulated in the FGF10‐treated blastemas at the whole transcriptome level. The top 10 upregulated genes are shown. Table S3. Genes that were downregulated in the FGF10‐treated blastemas at the whole transcriptome level. The top 10 downregulated genes are shown. Table S4. Downregulated DEGs contributing to each term in the enrichment analysis. Table S5. Upregulated DEGs with Fc >2 and their expression profiles. Table S6. Downregulated DEGs with Fc <0.5 and their expression profiles. Table S7. Numbers of cells in the previous report (Aztekin et al., 2021) that were projected to our data for annotation of corresponding clusters. [file DGD-64-266-s001.pdf]

Supporting Information

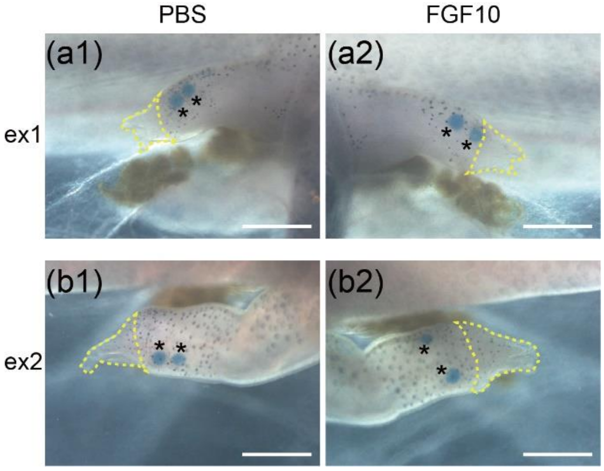

FIGURE S1. Morphologies of the PBS-treated and FGF10-treated blastemas. Hindlimbs of tadpoles at st. 56 were amputated and then implanted with PBS-soaked and FGF10-soaked beads near the amputated plane of right (a1, b1) and left (a2, b2) hindlimbs, respectively. (a, b) Two examples of implanted tadpoles (ex1 and ex2) at 7 dpa are shown. Asterisks indicate implanted beads. Yellow dotted lines indicate blastemas. Bars indicate 1 mm. The size of the FGF10-treated blastemas tended to be larger than that of the PBS-treated blastemas. Note that the photos show blastemas at 7 dpa when the size difference became clearer than that at 5 dpa, although blastemas at 5 dpa were used for the present study.

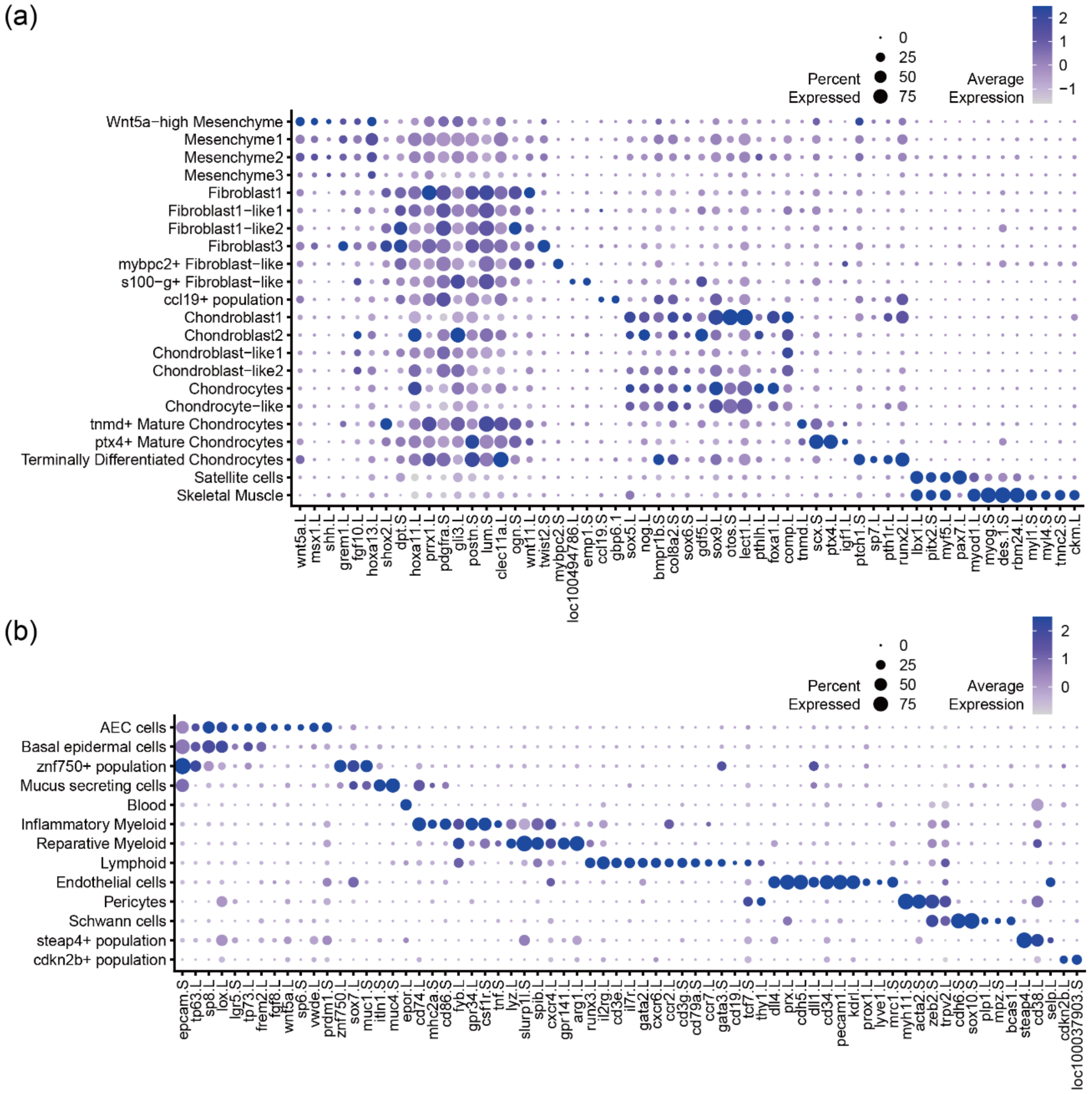

FIGURE S2. Expression profiles of various lineage marker genes and characteristic genes in mesenchymal and muscle (a), epidermal, blood, endothelial, and other (b) clusters. Average expression level is shown as z-score calculated using the mean expression value of each cluster in both conditions (PBS and FGF10) and of all cells in the displayed clusters, indicated by dot color depth. Percentage of expressing cells in each cluster is indicated by dot size. Mouse orthologues of *loc100494786.L* in (a) and *loc100037903.S* in (b) are *S100 calcium binding protein G (s100-g)* and *sulfotransferase family, cytosolic, 6B, member 1* (*sult6b1*), respectively.

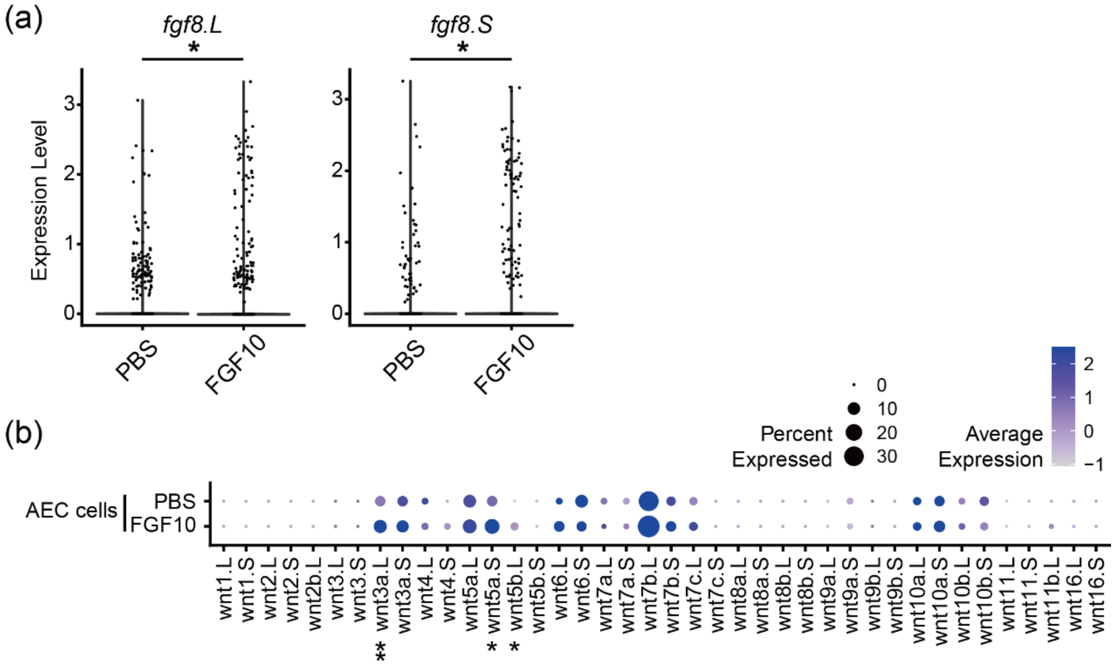

FIGURE S3. FGF10-treatment upregulated its downstream genes. (a) Violin plots of *fgf8.L* and *fgf8.S* expression in the PBS-treated and FGF10-treated samples. Normalized expression levels (transcripts per 10^4) in natural-log scale are shown. *P<0.01, Welch's *t*-test using normalized expression levels. (b) Expression levels of *wnt* genes in the AEC cell clusters of the PBS-treated and FGF10-treated samples. Average expression level is shown as z-score calculated using the mean expression value of the indicated cluster in each condition (PBS or FGF10) and of all cells, indicated by dot color depth. Percentage of expressing cells in each cluster is indicated by dot size. *P<0.05, **P<0.01, Welch's *t*-test using normalized expression levels.

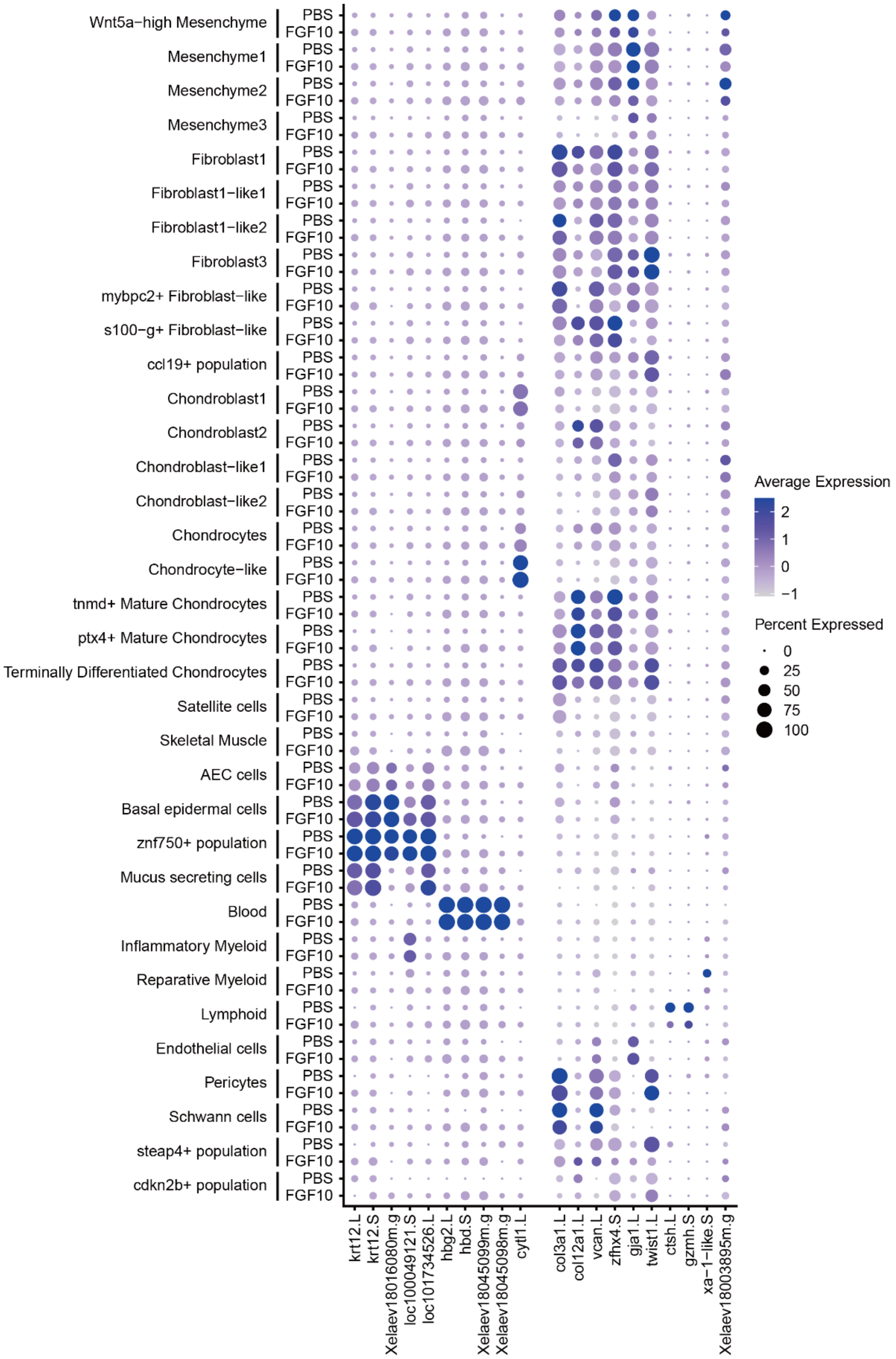

FIGURE S4. The top 10 upregulated and downregulated genes in the FGF10-treated blastemas at the whole transcriptome level. Average expression level is shown as z-score calculated using the mean expression value of the indicated cluster in each condition (PBS or FGF10) and of all cells, indicated by dot color depth. Percentage of expressing cells in each cluster is indicated by dot size.

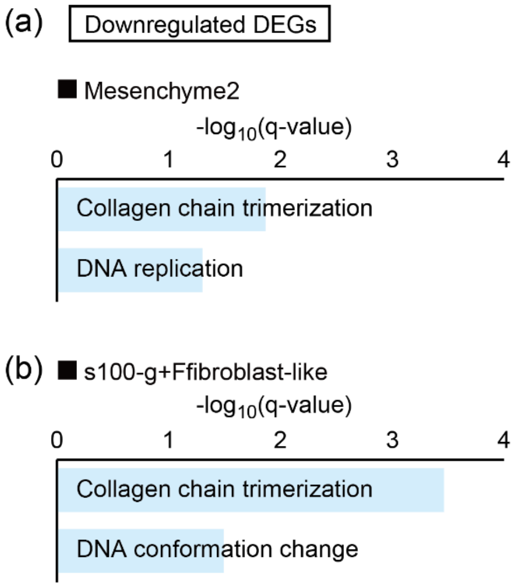

FIGURE S5. Enrichment analysis using mouse orthologues of downregulated DEGs detected in the Mesenchyme2 (a) and s100-g+ Fibroblast-like (b) clusters. Terms with $-\log_{10}$(q-value) > 1.3 (q-value < 0.05) are shown.

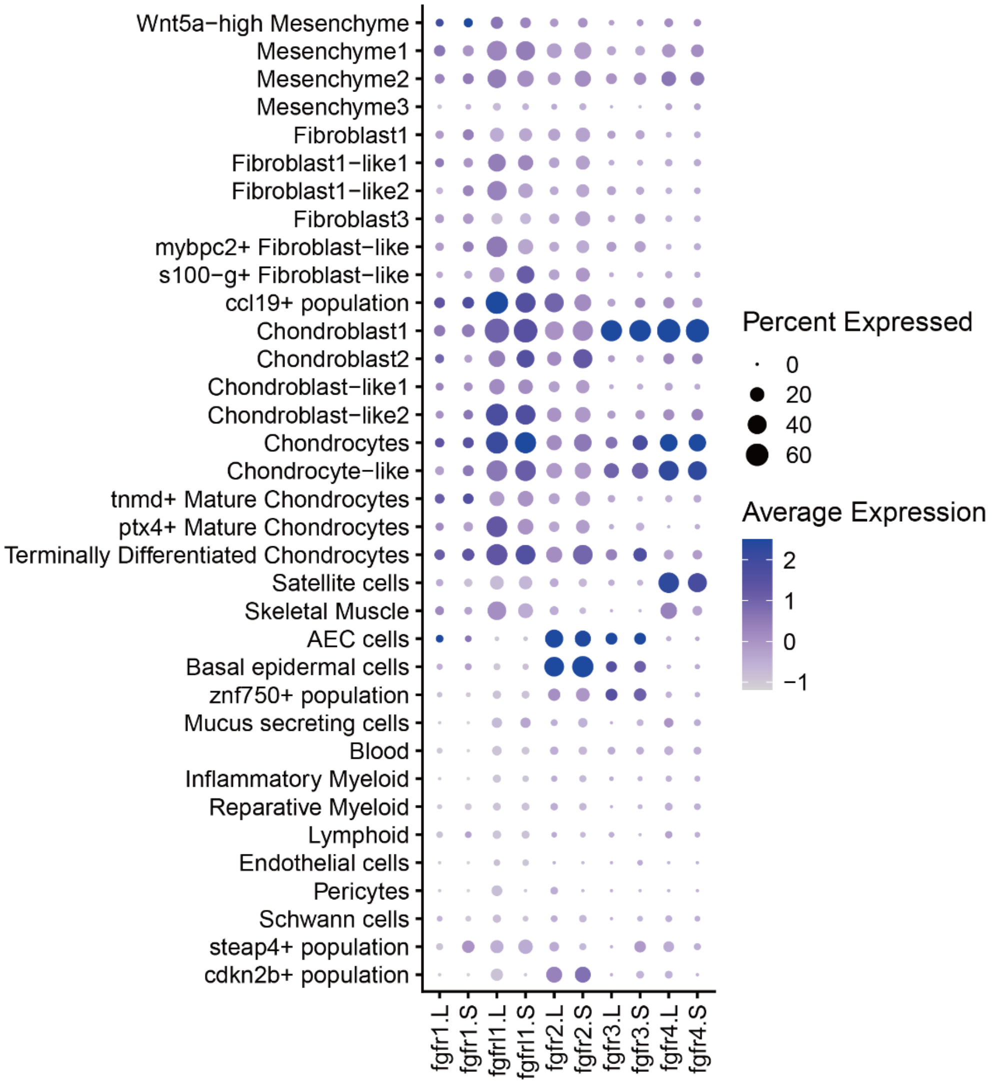

FIGURE S6. Expression of Fgf receptor genes in each cluster. Average expression level is shown as z-score calculated using the mean expression value of the indicated cluster in both conditions (PBS and FGF10) and of all cells, indicated by dot color depth. Percentage of expressing cells in each cluster is indicated by dot size. Human FGF10 was reported to interact with FGFR2, and weakly with FGFR1 (Zhang et al., 2006).

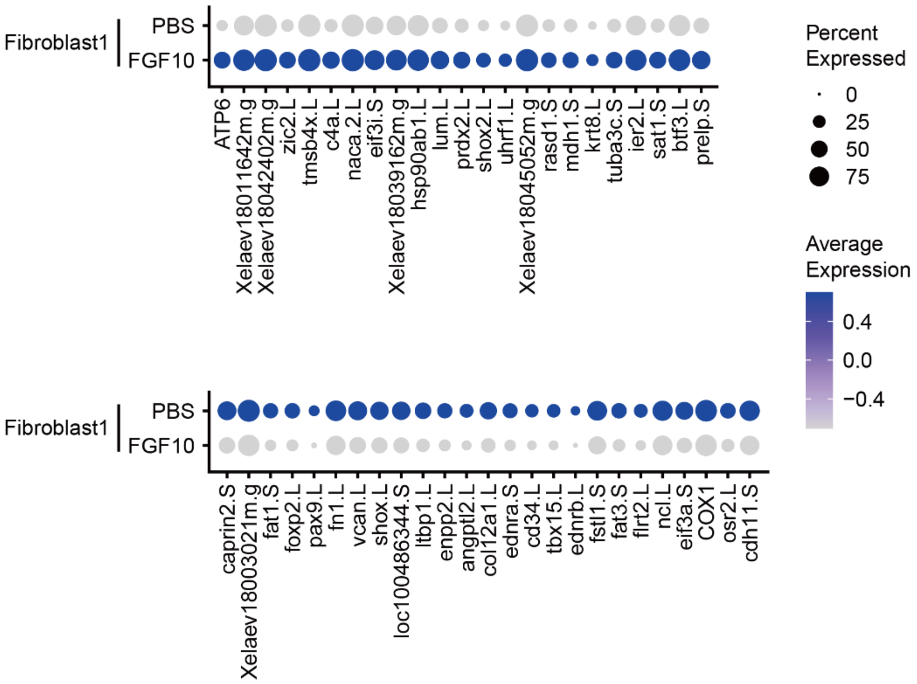

FIGURE S7. Comparison of the expression levels of upregulated and downregulated DEGs detected in the Fibroblast1 cluster. Average expression level is shown as z-score calculated with the mean expression value of cells in the cluster of each condition (PBS or FGF10) and of both conditions, indicated by dot color depth. Percentage of expressing cells in each cluster is indicated by dot size.

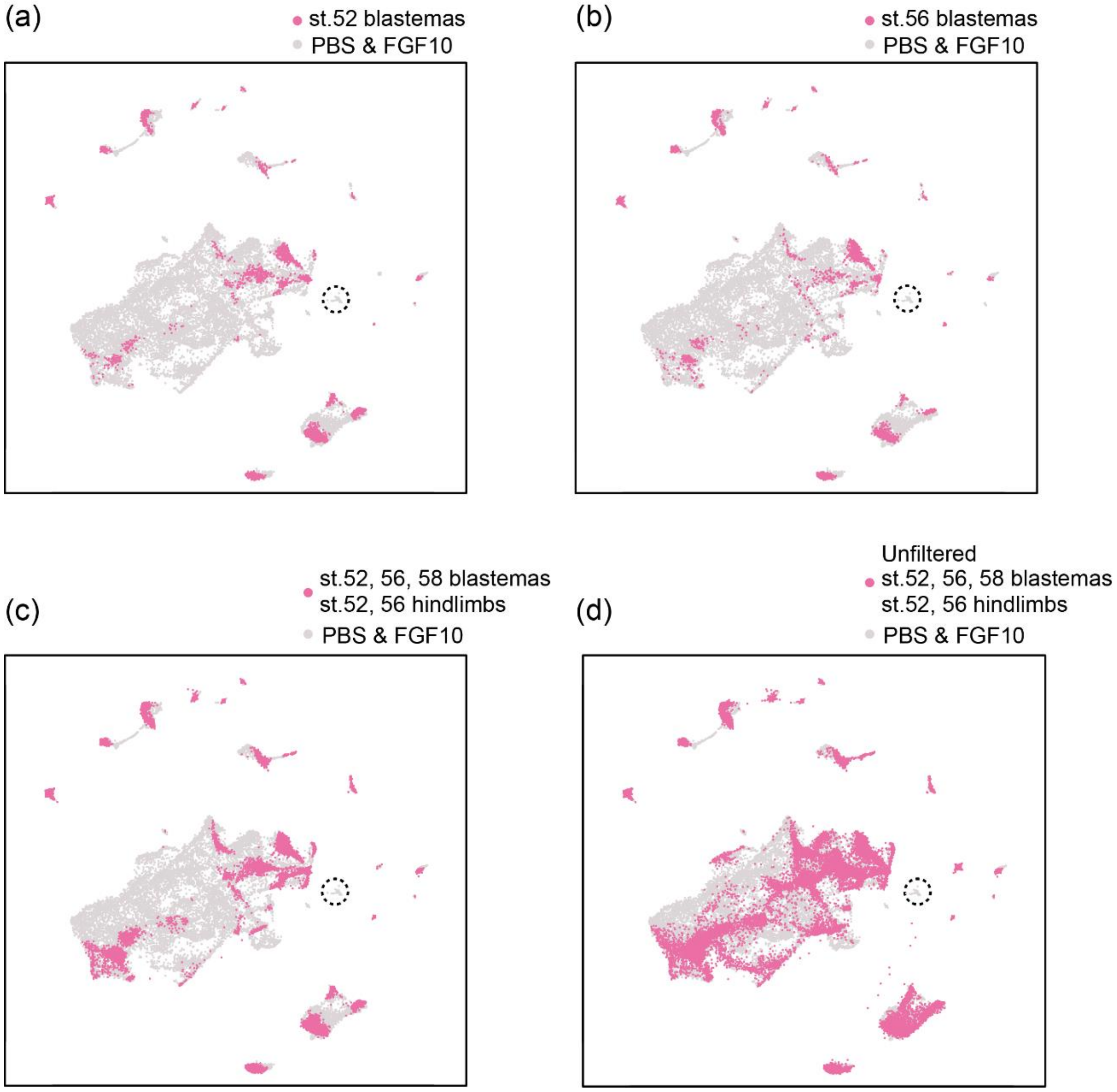

FIGURE S8. The scRNA-seq data of intact hindlimbs at st. 52 and 56, and blastemas of 5 dpa regenerating hindlimbs in tadpoles at st. 52, 56, and 58 reported previously (Aztekin et al., 2021) were projected to our scRNA-seq data to detect their corresponding clusters. The data of st. 52 tadpole blastemas (a), st. 56 tadpole blastemas (b), combined data of intact st.52 and 56 hindlimbs, and st. 52, 56, and 58 tadpole blastemas (c), combined data that were omitted the filtering process (excluding of cells with predicted.id.score < 0.9; low accuracy in identifying corresponding cluster) (d) (red dots) were projected to our data (gray dots) and visualized by a UMAP plot. No cells in the previous report were classified into the steap4+ population indicated by a broken line. Numbers of cells annotated into each cluster are shown in Table S7.

Table S1. Summary and quality control of the scRNA-seq data.

	PBS	FGF10
Total number of reads	488572059	415444901
% of reads mapped to genome	96.0 %	96.4 %
% of reads mapped confidently to transcriptome	64.7 %	66.3 %
Number of detected cells	7540	8266
Mean UMI counts per cell	10541	9233
Median UMI counts per cell	8173	7112
Mean detected genes per cell	2320	1968
Median detected genes per cell	2010	1778

Table S2. Genes that were upregulated in the FGF10-treated blastemas at the whole transcriptome level. The top 10 upregulated genes are shown.

Gene symbol	Fc†	pct.FGF10‡	pct.PBS‡	p_val_adj§
krt12.L	2.17	0.203	0.082	5.92E-99
hbg2.L	2.16	0.183	0.062	2.17E-114
hbd.S	2.12	0.208	0.088	5.08E-95
loc100049121.S¶	1.92	0.162	0.094	1.81E-36
krt12.S	1.91	0.207	0.094	1.61E-83
cytl1.L	1.88	0.245	0.139	1.61E-62
loc101734526.L¶	1.86	0.164	0.067	4.77E-75
Xelaev18045099m.g¶	1.81	0.199	0.08	6.64E-99
Xelaev18045098m.g¶	1.79	0.067	0.018	1.84E-46
Xelaev18016080m.g¶	1.76	0.122	0.054	8.88E-47

†Fc, Fold-change in the gene expression level; ‡pct.FGF10, pct.PBS, percentage of cells in which the gene was detected in the FGF10-treated and PBS-treated samples, respectively; §P_val_adj, adjusted *P*-value. ¶Mouse orthologues of loc100049121.S, loc101734526.L, Xelaev18045099m.g, Xelaev18045098m.g, and Xelaev18016080m.g are cystatin B, cystatin A1, hemoglobin X alpha-like embryonic chain in Hba complex, hemoglobin alpha adult chain 1, and keratin 5, respectively.

Table S3. Genes that were downregulated in the FGF10-treated blastemas at the whole transcriptome level. The top 10 downregulated genes are shown.

Gene symbol	Fc†	pct.FGF10‡	pct.PBS‡	p_val_adj§
ctsh.L	0.47	0.001	0.007	3.10E-04
gzmh.S	0.51	0.002	0.007	7.92E-03
xa-1-like.S¶	0.72	0.003	0.008	1.49E-02
col3a1.L	0.73	0.307	0.394	1.62E-25
vcan.L	0.77	0.316	0.426	2.16E-41
col12a1.L	0.77	0.152	0.232	7.13E-33
Xelaev18003895m.g¶	0.77	0.155	0.218	4.06E-22
gja1.L	0.78	0.196	0.268	2.33E-24
twist1.L	0.78	0.362	0.469	1.13E-44
zfhx4.S	0.78	0.41	0.529	4.11E-53

†Fc, Fold-change in the gene expression level; ‡pct.FGF10, pct.PBS, the percentage of cells in which the gene was detected in the FGF10-treated and PBS-treated samples, respectively; §P_val_adj, adjusted *P*-value. ¶No obvious orthologues were found in *Mus musculus* genes.

Table S4. Downregulated DEGs contributing to each term in the enrichment analysis.

**Extracellular matrix organization** R-MMU-1474244
Col11a1, Col12a1, Col14a1, Col3a1, Col5a1, Col6a1, Col6a2, Col1a1, Col1a2, Comp, Ctsk, Fbln2, Fbn2, Fn1, Itgb1, Mmp2, Plec, Sparc, Sdc1, Sdc3, Tgfb2, Tnc, Efemp2, Pxdn, Col16a1, Ltbp1, Emilin3, Col27a1, Crk, Postn, Ccdc80, Abi3bp, Flrt2, Actg1, Pdgfd, Ctnnb1, Ctnna1, Mdk, Fgfrl1, Cd34, Ifitm3, Atf4, Hsp90ab1, Foxo3, Wls, Tmem119, Anxa1, Arrdc3
**Regulation of cell-substrate adhesion** GO:0010810
Actg1, Calr, Col1a1, Crk, Emp2, Fbln2, Fn1, Itgb1, Lrp1, Mdk, Enpp2, Iqgap1, Postn, Dbn1, Efemp2, Ccdc80, Col16a1, Abi3bp, Efnb1, Hmgb1, Anxa1, Cyth3, Tgfb2, Foxo3, Mex3b, Zfp609, Mmp2, Tnc, Ednra, Cavin1, Sema3c, Sparc, Nipbl, Pdgfd
**Blood vessel development** GO:0001568
Actg1, Zfp36l1, Ctnnb1, Socs3, Col3a1, Col5a1, Col1a1, Col1a2, Comp, Ednra, Fn1, Gja1, Glul, Hand2, Itgb1, Junb, Lrp1, Mmp2, Myh9, Ncl, Notch2, Prrx1, Robo1, Sema3c, Tbx2, Tgfb2, Efemp2, Ltbp1, Col11a1, Col14a1, Vcan, Plec, Shox2, Nipbl, Foxp4, Fgfrl1, Asxl1, Flrt2, Tnc, Ednrb, Six4, Foxp2, Etv1, Zfp609, Cd34, H3f3a, Hmgb1, Dbn1, Zfp423, Osr2, Fat1, Wls
**Molecules associated with elastic fibres** R-MMU-2129379
Fbln2, Fbn2, Fn1, Itgb1, Tgfb2, Efemp2, Ltbp1, Emilin3, Cd34, Hsp90ab1, Cst3, Fstl1, Tnc, Mxra8, Vim
**Growth** GO:0040007
Zfp36l1, Ctnnb1, Col3a1, Comp, Ednra, Gas1, Gja1, H3f3a, Hsp90ab1, Itgb1, Anxa1, Mdk, Notch2, Plec, Robo1, Tbx2, Tgfb2, Tnc, Iqgap1, Postn, Foxo3, Nipbl, Foxp2, Septin7, Col27a1, Atf4, Cdh11, Efnb1, Etv1, Fat1, Fn1, Lrp1, Myh9, Ncam1, Sema3c, Top2b, Dbn1, Asxl1, Fat3, Flrt2, Actg1, Six4, Crk, Ednrb, Hmgb1, Enpp2, Ppia, Mmp2

Table S5. Upregulated DEGs with Fc > 2 and their expression profiles.

Cluster annotation	Gene symbol	Fc[†]	pct.FGF10[‡]	pct.PBS[‡]	p_val_adj[§]
Mesenchyme2	matn1.L	2.00	30.5	10.6	3.60E-05
Mesenchyme2	matn4.S	2.35	31.4	12.4	1.38E-04
Mesenchyme2	otos.S	2.29	29.2	10.6	2.97E-04
Mesenchyme3	COX2	2.43	47.0	23.2	1.96E-02
Fibroblast1-like1	hbd.S	2.10	21.8	6.8	5.38E-05
AEC cells	fos.S	3.32	74.6	46.2	4.45E-06
AEC cells	socs3.S	2.49	58.8	25.3	1.39E-03
AEC cells	id3.L	2.68	65.6	39.6	1.82E-03
AEC cells	ier5l.S	2.53	58.8	30.8	7.82E-03
AEC cells	junb.L	2.24	73.2	50.5	3.96E-02
Basal epidermal cells	fos.S	2.09	88.1	69.9	4.73E-16
Basal epidermal cells	junb.L	2.07	82.8	62.5	9.26E-11
Basal epidermal cells	jun.S	2.16	58.5	38.2	6.91E-07
Basal epidermal cells	socs3.S	2.09	56.0	36.3	3.70E-06
Basal epidermal cells	loc100049121.S[¶]	2.09	60.5	42.5	1.72E-04
Reparative Myeloid	mmp1.S	9.39	59.2	26.5	4.24E-04
Reparative Myeloid	ncf1.L	2.88	77.7	51.3	7.13E-04
Reparative Myeloid	cnfn.1.S	6.76	43.7	12.4	9.13E-04
Reparative Myeloid	irg1.L	4.73	42.7	15.0	1.06E-02
Reparative Myeloid	cyba.L	2.04	88.3	79.6	2.59E-02
Reparative Myeloid	loc100489423.L[¶]	4.11	37.9	10.6	3.43E-02
Reparative Myeloid	il1b.S	2.96	52.4	23.9	3.82E-02
Reparative Myeloid	prtn3.S	4.41	42.7	14.2	4.72E-02
steap4+ population	chit1.S	13.45	73.3	0.0	6.81E-03
steap4+ population	mmp8.L	17.05	100.0	50.0	7.32E-03

[†]Fc, Fold change of gene expression level; [‡]pct.FGF10, pct.PBS, the percentage of cells where the gene is detected in the cluster of the FGF10-treated and PBS-treated samples, respectively; [§]P_val_adj, adjusted *P*-value. [¶]The mouse orthologues of *loc100049121.S* and *loc100489423.L* are *cystatin B* and *toll-like receptor 5,* respectively.

Table S6. Downregulated DEGs with Fc < 0.5 and their expression profiles.

Cluster annotation	Gene symbol	Fc†	pct.FGF10‡	pct.PBS‡	p_val_adj§
Chondrocytes	Xelaev18003021m.g¶	0.45	86.8	89.6	4.29E-05
Fibroblast1	enpp2.L	0.43	21.1	39.9	1.11E-04
Mesenchyme2	Xelaev18003895m.g¶	0.46	28.0	48.1	3.55E-03
s100-g+Ffibroblast-like	socs3.S	0.47	45.3	73.8	1.92E-07
s100-g+Ffibroblast-like	enpp2.L	0.40	17.2	41.9	1.93E-04
s100-g+Ffibroblast-like	Xelaev18003021m.g¶	0.46	89.2	96.4	3.93E-02
Chondroblast-like2	comp.L	0.48	25.0	57.4	8.80E-06
tnmd+ Mature Chondrocytes	cdo1.L	0.50	16.3	58.1	4.23E-02

†Fc, Fold change of gene expression level; ‡pct.FGF10, pct.PBS, the percentage of cells where the gene is detected in the cluster of the FGF10-treated and PBS-treated samples, respectively; §P_val_adj, adjusted *P*-value. ¶No obvious orthologues were found in *Mus musculus* genes.

Table S7. Numbers of cells in the previous report (Aztekin et al., 2021) that were projected to our data for annotation of corresponding clusters. The data of the tadpole blastemas of 5 dpa regenerating hindlimbs at st. 52, 56, and 58 (blas), and control intact hindlimbs of tadpoles at st. 52 and 56 (cont) in the previous report were used for analysis. Cells with predicted.id.score < 0.9 (low accuracy in identifying corresponding cluster) were excluded from the analysis.

Cluster annotation	st52_blas	st52_cont	st56_blas	st56_cont	st58_blas
Wnt5a-high Mesenchyme	0	0	0	0	0
Mesenchyme1	273	165	111	6	109
Mesenchyme2	186	109	147	170	156
Mesenchyme3	310	98	587	19	118
Fibroblast1	18	11	39	34	13
Fibroblast1-like1	1	0	1	0	0
Fibroblast1-like2	1	0	20	20	18
Fibroblast3	33	15	46	53	62
mybpc2+ Fibroblast-like	0	0	0	0	0
s100-g+Ffibroblast-like	0	0	0	0	0
ccl19+ population	0	0	0	0	1
Chondroblast1	88	35	126	215	640
Chondroblast2	12	4	15	29	11
Chondroblast-like1	0	0	0	0	0
Chondroblast-like2	0	0	0	0	0
Chondrocytes	44	31	53	62	93
Chondrocyte-like	0	0	0	0	0
tnmd+ Mature Chondrocytes	0	0	0	0	0
ptx4+ Mature Chondrocytes	0	0	4	8	2
Terminally Differentiated Chondrocytes	0	1	8	31	14
Satellite cells	79	111	57	118	78
Skeletal Muscle	12	18	33	23	14
AEC cells	499	94	108	44	50
Basal epidermal cells	946	441	319	363	342
znf750+ population	370	207	583	390	1050
Mucus secreting cells	0	0	3	0	0
Blood	741	1348	626	987	599
Inflammatory Myeloid	225	53	281	62	235
Reparative Myeloid	255	15	877	116	341
Lymphoid	80	43	129	91	180
Endothelial cells	34	12	23	20	53
Pericytes	14	4	18	16	25
Glial cells	13	10	15	25	53
steap4+ population	0	0	0	0	0
cdkn2b+ population	0	0	0	0	0

References

Aztekin, C., Hiscock, T.W., Gurdon, J., Jullien, J., Marioni, J., & Simons, B.D. (2021). Secreted inhibitors drive the loss of regeneration competence in Xenopus limbs. *Development* **148**. dev199158. http://doi.org/10.1242/dev.199158.

Zhang, X., Ibrahimi, O. A., Olsen, S. K., Umemori, H., Mohammadi, M., & Ornitz, D. M. (2006). Receptor specificity of the fibroblast growth factor family. The complete mammalian FGF family. *The Journal of biological chemistry*, 281(23), 15694–15700. https://doi.org/10.1074/jbc.M601252200.
